# Experimental evolution of a more restrained clutch size when filial cannibalism is prevented in burying beetles *Nicrophorus vespilloides*

**DOI:** 10.1002/ece3.8829

**Published:** 2022-04-15

**Authors:** Darren Rebar, Chay Halliwell, Rachel Kemp, Rebecca M. Kilner

**Affiliations:** ^1^ 3218 Department of Biological Sciences Emporia State University Emporia Kansas USA; ^2^ 2152 Department of Zoology University of Cambridge Cambridge UK; ^3^ Department of Animal and Plant Sciences University of Sheffield Sheffield UK

**Keywords:** lack clutch size, optimistic clutch size, parental care, plasticity, reaction norm

## Abstract

The overproduction of offspring is commonly associated with high hatching failure and a mechanism for dispensing with surplus young. We used experimental evolution of burying beetle populations *Nicrophorus vespilloides* to determine causality in these correlations. We asked does eliminating the mechanism for killing “spare” offspring cause the evolution of a more restrained clutch size and consequently select for reduced hatching failure? *N*. *vespilloides* typically overproduces eggs but kills 1^st^ instar larvae through partial filial cannibalism during brood care. We established replicate evolving populations that either could practice filial cannibalism (Full Care) or could not, by removing parents before their young hatched (No Care). After 20+ generations of experimental evolution, we measured clutch size and hatching success. We found that No Care females produced fewer eggs than Full Care females when allowed to breed on a small corpse, a finding not explained by differences in female quality. On larger corpses, females from both populations laid similar numbers of eggs. Furthermore, hatching success was greater in the No Care populations on small corpses. Our results suggest that the adaptive overproduction of offspring depends on a mechanism for eliminating surplus young and that killing offspring, in turn, relaxes selection against hatching failure.

## Graphical abstract

The overproduction of offspring is commonly associated with high hatching failure and a mechanism for dispensing with surplus young. We used experimental evolution of burying beetle populations *Nicrophorus vespilloides* to ask whether eliminating the mechanism for killing “spare” offspring caused the evolution of a more restrained clutch size and consequently select for reduced hatching failure. Our results suggest that the adaptive overproduction of offspring depends on a mechanism for eliminating surplus young and that killing offspring, in turn, relaxes selection against hatching failure.

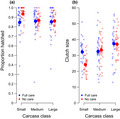

## 1 INTRODUCTION

Multicellular organisms typically produce many more offspring than will ever mature into reproducing adults (Forbes, 2005). The overproduction of offspring is commonly correlated with high levels of uncertainty about the likely success of the breeding attempt and a mechanism for killing any spare offspring (Mock & Parker, 1997). But which is the more important causal agent in driving the production of excess young: uncertainty about reproductive success or a mechanism for eliminating surplus offspring?

The classic adaptive explanation is that uncertainty about the success of the breeding attempt is the key factor driving the overproduction of offspring (Forbes, 1991, 1997; Kozlowski & Stearns, 1989; Lack, 1947; Magrath, 1989; Mock & Forbes, 1995; Mock & Parker, 1997; Ricklefs, 1965; Temme & Charnov, 1987). Uncertainty may derive from extrinsic sources, such as the resources available to nourish dependent young (Kozlowski & Stearns, 1989; Lack, 1947; Ricklefs, 1965; Temme & Charnov, 1987) or the likelihood that offspring will be attacked by rivals (Giron et al., 2004). There is additional uncertainty about the intrinsic qualities of each unborn offspring (Forbes, 1991, 1997). Some may fail to hatch, for example, or develop abnormally due to genetic mutation.

The classic explanation suggests that an “optimistic” strategy, involving the production of more offspring than can ever be raised to independence, provides an adaptive way for parents to maximize their fitness in the face of these unknowns. Surplus individual offspring are then actively, or passively, eliminated to achieve the optimal family size for the conditions that then actually prevail (Forbes, 1991, 1997; Kozlowski & Stearns, 1989; Magrath, 1989; Mock & Forbes, 1995; Mock & Parker, 1997; Ricklefs, 1965; Temme & Charnov, 1987).

More recent work, however, suggests that a mechanism for cheaply dispensing with extra young may be more important in driving the evolution of super‐fecundity. The rationale is that the overproduction of offspring can only evolve if the net fitness benefits of producing too many offspring, and then eliminating them, are greater than the net fitness payoffs from producing far fewer offspring (Forbes, 1990, 1991; Forbes & Mock, 1998; Houston et al., 2012). Unless these conditions are met, the overproduction of offspring cannot be favored by selection—no matter how uncertain the prospects for successful reproduction are.

One corollary of the overproduction of offspring that has received relatively little consideration is that it relaxes selection against sources of uncertainty that are due to intrinsic causes, such as hatching failure or the phenotypic expression of genetic mutations. Once parents start producing surplus young then the costs of losing individual offspring to intrinsic causes of failure, such as hatching failure, are more easily borne (Forbes, 1991). What does the death of a single offspring matter if it is just one among many and was never going to be raised to independence anyway? Thus, the overproduction of offspring weakens selection against intrinsic causes of failure, like hatching failure. Mechanistically, if eggs are laid in large quantities then hatching failure may persist because each egg receives relatively few resources and some may have insufficient resources for the embryo within to complete development successfully.

Here, we test these ideas using experimental evolution of burying beetles *Nicrophorus vespilloides*, while holding most forms of uncertainty about breeding success as constant as possible. We manipulated the mechanism for eliminating surplus offspring (so that it was consistently present in some populations and consistently absent in others, for generation after generation) and determined the evolutionary consequences for clutch size and hatching failure. In common with many other taxa, *N*. *vespilloides* parents kill surplus offspring through partial filial cannibalism (Klug & Bonsall, 2007; Manica, 2002). Partial filial cannibalism potentially increases the fitness of remaining offspring when resources are limiting (Bartlett, 1987; Schrader et al., 2015a; Trumbo & Fernandez, 1995) because there is a marked trade‐off between brood size and larval size at dispersal in burying beetles. Eliminating some offspring thus promotes larval mass at dispersal, which is a strong predictor of future fitness (Bladon et al., 2020; Lock et al., 2004; Pascoal et al., 2018) while also providing an energetic benefit to parents.

Our analyses test whether the loss of filial cannibalism favors the evolution of a more restrained clutch size, and whether a more restrained clutch size in turn selects for increased hatching success because the fate of individual offspring then makes a relatively greater contribution to parental fitness. Specifically, we predicted that (1) we should see selection for reduced clutch size in a No Care environment. Consequently, after several generations of experimental evolution: (2) No Care populations without filial cannibalism should produce a more restrained clutch size than Full Care populations with filial cannibalism, especially when resources are limited. In addition, (3) clutches produced by No Care populations without filial cannibalism should have greater hatching success than clutches produced by Full Care populations with filial cannibalism.

## 2 METHODS

### 2.1 Burying beetle natural history

Burying beetles *N*. *vespilloides* breed upon the dead body of a small vertebrate, such as a songbird or rodent. They strip the body of fur or feathers, roll the flesh into a ball, and then bury it below ground where it becomes an edible nest for their larvae (Scott, 1998; Sikes et al., 2002). Egg laying takes place during carcass preparation, and larvae hatch in the soil where the eggs are laid before crawling to the edible nest made from the carcass by their parents. Both parents tend to their young after hatching but larvae can self‐feed too (Smiseth et al., 2003) and are capable of surviving with no post‐hatching care at all (Rebar et al., 2020).

Females crudely adjust the number of eggs they lay to match the size of the dead body and generally lay more eggs when breeding upon a larger corpse (Müller et al., 1990; Trumbo & Fernandez, 1995). After hatching, brood size is more carefully matched to the resources upon the carcass through partial filial cannibalism, when parents consume excess 1^st^ instar larvae (Bartlett, 1987; Müller, Eggert, & Furlkröger, 1990; Trumbo & Fernandez, 1995). Parents have been found to consume up to half of the larvae to optimize the brood size to the carcass (Bartlett, 1987), and brood size has an important bearing on the fitness attained by individual larvae. Larvae that develop in larger broods are smaller by the time they disperse away into the soil to pupate because the finite resources on the carcass are then spread more thinly among individual offspring. Smaller larvae have reduced survival and develop into smaller adults (Lock et al., 2004), with lower fecundity (Pascoal et al., 2018; Safryn & Scott, 2000; Schrader et al., 2016; Scott, 1998). Indeed, adult body size is almost entirely dependent on the nutritional environment experienced during development and has close to zero heritability (Jarrett et al., 2017).

### 2.2 Populations subjected to experimental evolution

Details of the experimentally evolving populations of *N*. *vespilloides*, including the methods by which they were established, are given in Jarrett, Evans, et al. (2018) and Rebar et al. (2020). In brief, wild‐caught beetles from different sites were interbred to create a genetically diverse stock population from which the populations subjected to experimental evolution were derived. It is possible that the founding population thus harbored greater potential to respond rapidly to selection than is typically present in each wild population. Four experimental populations were established, two with full post‐hatching parental care, Full Care, and the other two without any post‐hatching parental care, No Care. For logistic simplicity, the replicate populations were organized into two blocks (Block 1 and Block 2, referred to below in the statistical analyses), with one Full Care and one No Care population within each block. In Full Care populations, parents were able to practice partial filial cannibalism. In No Care populations, they were not. (To our knowledge, parents do not practice ovicide on their own eggs in *N*. *vespilloides*.)

In the Full Care populations, a minimum of 30 pairs of unrelated beetles were bred each generation at 17 days post‐adult eclosion. Pairs were randomly assigned, excluding sibling and cousin pairings. Each pair was placed in a breeding box (17 × 12 × 6 cm) containing moistened soil and a thawed mouse carcass (8–14 g). Breeding boxes were stored in a dark cabinet to simulate the natural underground environment. Parents remained in the box throughout larval development, allowing the full suite of parent‐offspring interactions to be expressed. Eight days after pairing, the dispersing larvae from each family were placed into individual cells (2 cm^3^) in a box containing 5 × 5 cells, covered with moistened peat, and left undisturbed to pupate into adults. Newly eclosed adults were housed individually until breeding, a minimum of 17 days after eclosion. All adult beetles were fed ca. 1 g raw ground beef twice a week.

In the No Care populations, the same protocol as above was followed, but with two exceptions. First, both parents were removed from the breeding box 53 h after pairing, which allowed parents to prepare the carcass and females to lay eggs but prevented any post‐hatching parental care (Boncoraglio & Kilner, 2012; Smiseth et al., 2006). Second, a minimum of 50 pairs of unrelated beetles were bred for the first 15 generations to offset the increased number of broods that failed (i.e., no larvae survive to dispersal, see Schrader et al., 2017). The effective population size at each generation was thus similar for both the No Care and Full Care populations. For both Full Care and No Care populations, adults were only allowed to breed once, soon after reaching sexual maturity. Thus, under the conditions of our experiment, the primary beneficiaries of filial cannibalism were the offspring (through the changed trade‐off between brood size and larval size) rather than the parents who had little capacity to gain fitness through any resources derived from filial cannibalism.

We have previously reported the evolution of both offspring and parental traits in these evolving populations, following sustained exposure to No Care and Full Care environments (Duarte et al., 2021; Jarrett, Rebar, et al., 2018; Rebar et al., 2020; Schrader et al., 2015a, 2017). For the work reported here, we harvested newly eclosed adults from the Full Care and No Care populations from generations 20 and 22 for use in predictions 2 and 3, respectively. We housed all beetles individually until they were bred for each experiment.

### 2.3 Prediction 1: Is there selection for smaller clutches in the absence of post‐hatching parental care?

We estimated the strength of selection on clutch size in the absence of post‐hatching parental care in relation to two aspects of fitness: (i) the number of larvae at dispersal (i.e., brood size) and (ii) the average mass of each larva in the brood at dispersal (i.e., mean larval mass) because average larval mass at dispersal predicts future fecundity and survival in both males and females (Bladon et al., 2020). To estimate selection, we used part of the dataset collected on the Full Care populations in the experiments described in Duarte et al. (2021) and focused only on generation 13. Briefly, in generation 13 adult males and females from the Full Care experimental populations were randomly paired, placed in a breeding box, and given an 11–14 g thawed mouse carcass (*n* = 25 pairs per population used here for analysis). Parents were then removed after 53 h, before any larvae hatched. At that time, the number of eggs visible on the bottom of the breeding box was counted, which is a non‐invasive way to deduce clutch size (Schrader et al., 2016). Carcasses were also swapped between breeding boxes, meaning that larvae developed on a carcass prepared by unrelated adults and in the absence of receiving any post‐hatching parental care as parents had already been removed. After 8 d, the remaining larvae were counted and weighed to the nearest 0.1 mg.

#### 2.3.1 Statistical analysis

We used R 4.0.2 (R Core Team, 2020) to regress the relative fitness of brood size and mean larval mass on standardized clutch size in separate linear models. Owing to slight differences between the two replicated Full Care populations (see Duarte et al., 2021), we calculated our two measures of relative fitness and standardized clutch size with respect to the population. We thus divided each individual trait value (brood size and mean larval mass) by the population mean of that trait value to generate the relative fitness (mean = 1). For standardizing clutch size (mean = 0 and SD = 1), we subtracted the population mean from the clutch laid by each female and divided that value by the population standard deviation. We then merged the two sets of population values into one dataset to perform the regressions, and we retained the failed broods (*n* = 9) for the brood size estimate. We measured direct selection on clutch size by estimating the selection gradient (*β*) from the slope of the regression between each relative fitness measure and clutch size (Brodie et al., 1995; Linnen & Hoekstra, 2009).

### 2.4 Prediction 2: Do No Care females match their clutch size to carcass size more effectively than Full Care females?

We randomly paired 40 males and females, from each of the four populations (*n* = 160 pairs in total), when they reached sexual maturity 17 days post eclosion, in a standard breeding box (17 × 12 × 6 cm) and gave them a dead mouse to breed upon. Mouse carcasses ranged from 8–24 g. To ensure that each population received a similar distribution of carcass weights, we subdivided carcasses into 2 g weight categories (i.e., 8–10 g and 10–12 g) and allocated five mice from each 2 g range to each population. Carcasses were then allocated haphazardly to a pair of beetles and each pair was placed with their mouse in a breeding box partially filled with moistened soil. We put each breeding box in a dark cabinet to simulate underground conditions and allowed the beetles to prepare the carcass and the female to lay a clutch of eggs.

After 53 h, we removed the parents from each breeding box and measured their pronotum width, a widely used proxy for size in burying beetles (Müller et al., 1990; Trumbo, 1992), to the nearest 0.01 mm with digital calipers (CD‐6″ ASX; Mitutoyo Corp.). We discarded the carcass and emptied the contents of each box into a tray. We then carefully sifted through the soil to count the number of eggs that each female deposited in the soil.

#### 2.4.1 Statistical analysis

We used R 4.0.2 (R Core Team, 2020) to perform a linear model with clutch size as the dependent variable. We initially included quadratic and linear terms for carcass mass, along with their interaction with population (Full Care or No Care), to explore the relationship between clutch size and carcass mass. We also included female size and male size as covariates and a term for block as a fixed effect to account for the replicated populations. We did not find support for a nonlinear relationship between clutch size and carcass mass and thus removed those terms from the model. We also did not find that male size explained variation in clutch size and subsequently removed it. Thus, the final model included terms for population and carcass mass, their interaction, and block, and female size as a covariate.

Given a significant effect of block in the model (see Table 1), we separated the two blocks to analyze whether female adjustments to clutch size were similar in each replicated experimental population. The models used in these analyses were identical to the initial model described above, minus a term for block. Table S1 and Figure S1 present those models and the raw data, respectively. The results show a similar pattern of evolutionary change across the replicated experimental populations. We thus report the overall model with block included in the results.

### 2.5 Prediction 3: Do eggs laid by No Care females have greater hatching success than those laid by Full Care females?

In a further experiment, performed two generations after the experiment testing Prediction 2, we compared the hatching success of clutches laid by females from the Full Care and No Care populations. We paired 45 males and females from each population (*n* = 180 pairs in total) at 17 days posteclosion, placing each pair in their own breeding box with moist soil and a thawed carcass. Pairs were randomly given a carcass from one of three size classes: small (8–9 g), medium (16–17 g), or large (23–24 g), spanning the range in carcass size used in the experiment to test Prediction 2. We haphazardly assigned 15 pairs per carcass category per population. We then placed each breeding box in a dark cabinet and allowed parents 53 h to prepare the carcass and for the female to lay a clutch of eggs. We removed parents and measured their pronotum width to the nearest 0.01 mm and then discarded the carcass.

We carefully sifted through the soil in each breeding box to remove the entire clutch of eggs. To assess hatching success without any confounding effects of the No Care/Full Care treatments, we transferred each clutch to its own dedicated petri dish lined with a 1.5% agar solution dissolved in PBS. Petri dishes were kept in darkness, and we checked for newly hatched larvae every 4 h, starting at 56 h after pairing. We removed larvae from the petri dishes each time they were checked to prevent larvae from damaging the remaining eggs. After 110 h of checking, any remaining eggs were scored as failing to hatch.

#### 2.5.1 Statistical analysis

We used R 4.0.2 (R Core Team, 2020) to perform a generalized linear model with a binomial distribution and logit link function and with the number of hatched versus unhatched eggs as the dependent variable. We removed clutches in which no eggs successfully hatched (*n* = 5), owing to uncertainty in the cause of failure. The final model included terms for population and carcass size class, along with their interaction. We also included clutch size as a covariate and a term for block as a fixed effect to account for the replicated populations. We initially included male size as covariate, but it did not contribute to the model and was thus removed.

We found a significant block effect in the model (see Table 2), prompting us to separate the two blocks to analyze whether the response was similar across populations. The terms used in these models were identical to the initial model described above, minus a term for block. Table S2 and Figure S2 show that the replicate experimental populations evolved in a similar way, and we thus report the overall model with block included in the results.

We used the data from this experiment to also test whether the effect of carcass mass on clutch size remained in the experimental populations. We performed a linear model with clutch size as the dependent variable. The final model included terms for population and carcass class, along with their interaction. We also included female size as a covariate and a term for block as a fixed effect to account for the replicated populations.

As with our previous models, we found a significant effect of block (see Table 2) and thus separated the blocks to analyze clutch size separately for each block. The terms used in these models were identical to the initial model described above, minus a term for block. Table S3 and Figure S3 show that the replicate experimental populations evolved in a similar way, and we thus report the overall model with block included in the results.

## 3 RESULTS

### 3.1 Prediction 1: Is there selection for smaller clutches in the absence of post‐hatching parental care?

**FIGURE 1:**
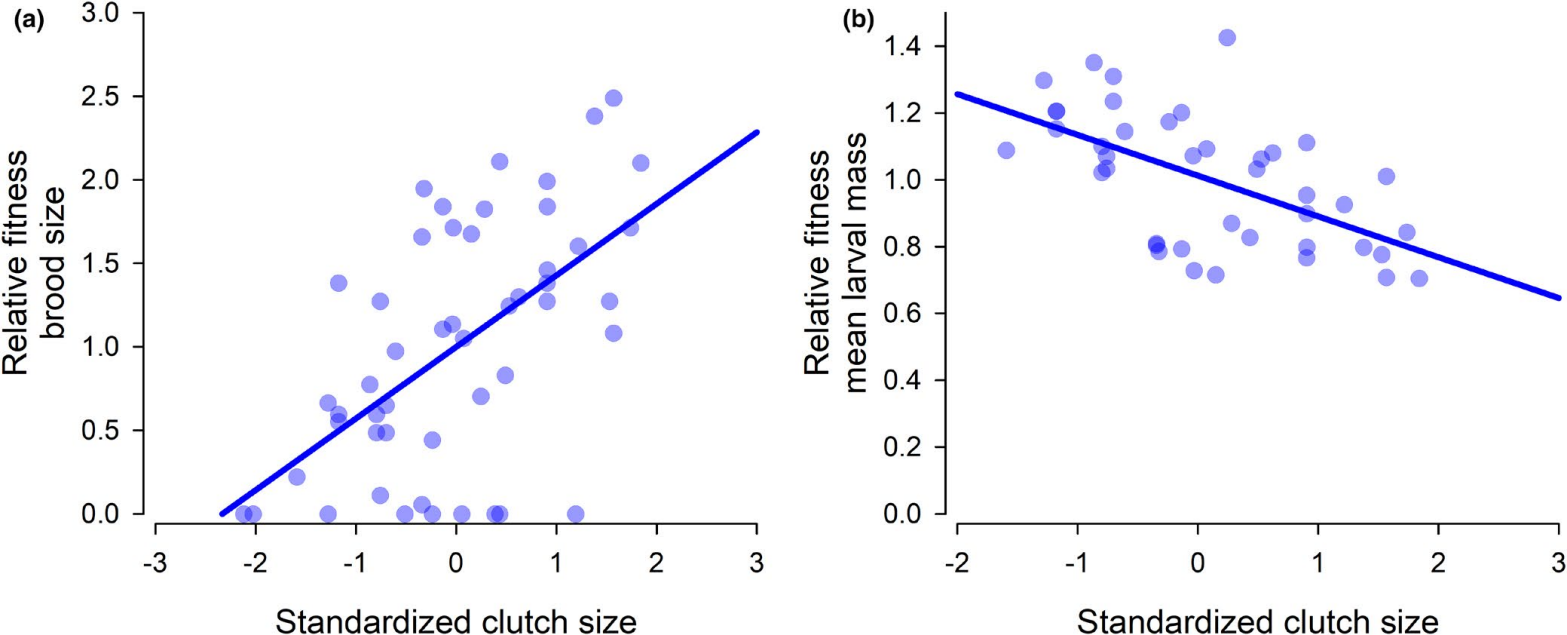
The relative fitness of (a) brood size at dispersal and (b) mean larval mass at dispersal in relation to standardized clutch size of offspring from generation 13 of the Full Care experimental populations but reared without any post‐hatching parental care. Offspring developed on a carcass prepared by a donor pair of Full Care beetles until dispersal. The slopes of the lines denote the strength of direct selection (estimated through the selection gradient; *β*). Data were collected in some of the experiments described in Duarte et al. (2021)

The two measures of fitness, brood size at dispersal and mean larval mass at dispersal, were strongly correlated with clutch size in the Full Care populations at generation 13 when offspring developed without post‐hatching parental care (Figure 1), but the selection gradients (i.e., slope; *β*) had opposing signs. There was selection on females to lay larger clutches of eggs because more larvae survived to dispersal (linear regression: *F*_1, 48_ = 23.49, *p* < .001, *β* = 0.428; Figure 1a). However, selection also favored offspring from smaller clutches because they yielded larger larvae at dispersal, with greater fitness (linear regression: *F*_1, 39_ = 19.62, *p* < .001, *β* = −0.122; Figure 1b).

### 3.2 Prediction 2: Do No Care females match their clutch size to carcass size more effectively than Full Care females?

**FIGURE 2:**
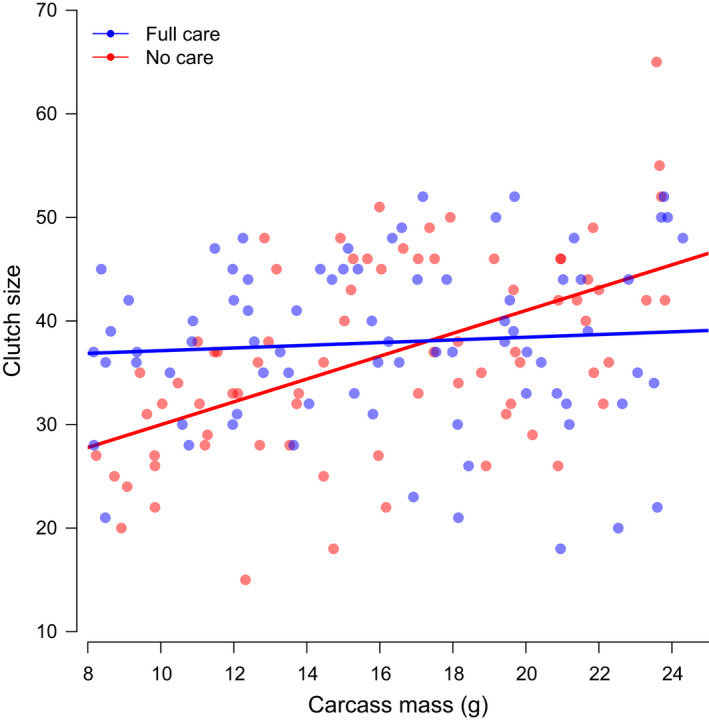
The relationship between clutch size and carcass mass for females that evolved in the Full care (blue) and No care (red) experimental populations. Lines show linear regression lines fitted through data collected from both replicates of each type of experimental population

We found that the relationship between carcass size and clutch size was significantly different for females from the Full Care versus No Care populations (Figure 2, Table 1). Specifically, No Care females were more sensitive to changes in carcass mass. They laid fewer eggs than Full Care females when given a small carcass and laid more eggs than Full Care females when given a large carcass (Figure 2), the latter partially driven by one No Care female producing an exceedingly large clutch of eggs. However, we note that removal of this datapoint did not qualitatively change our findings. By contrast, Full Care females generally produced a similar number of eggs regardless of the size of the dead mouse they were given to breed upon (Figure 2).

**TABLE 1:** Estimates from a linear model of the influence of carcass mass on the size of clutches laid by Full Care (FC) and No Care (NC) females

Clutch size	Estimate	Std error	*t* Value	*p*
Intercept	2.3041	7.2340	0.319	.751
Population (NC)	−12.1157	4.2632	**−2.842**	.**005**
Carcass mass	0.1664	0.1693	0.983	.327
Population (NC) × Carcass mass	0.8107	0.2508	**3.232**	.**002**
Female size	6.4530	1.4249	**4.529**	**<.001**
Block (2)	4.9556	1.1430	**4.336**	**<.001**

Note

Sample sizes were as follows 39 Full Care and 38 No Care in Block 1; 40 Full Care and 37 No Care in Block 2. Significant terms are in bold.

### 3.3 Prediction 3: Do eggs laid by No Care females have greater hatching success than those laid by Full Care females?

**FIGURE 3:**
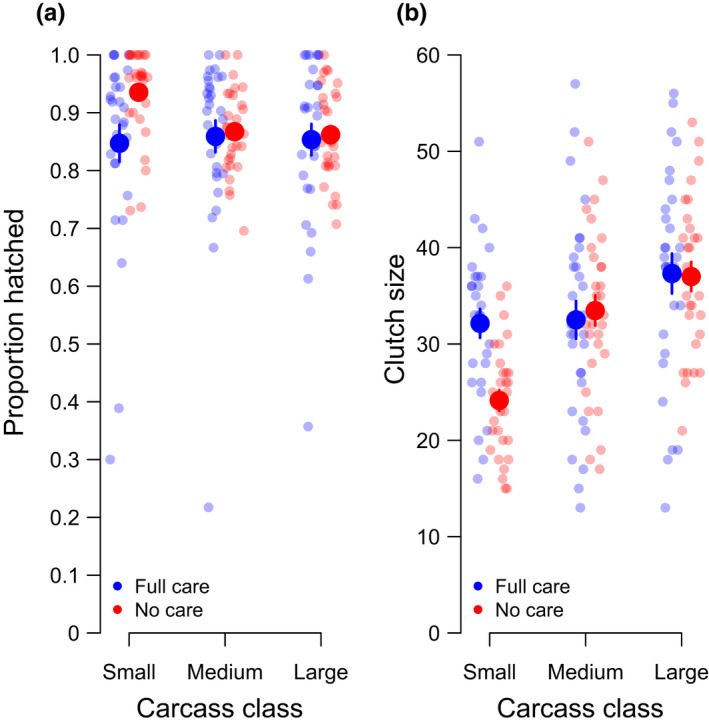
The (a) proportion of eggs that hatched and (b) size of clutches laid by Full Care (blue) and No Care (red) females across three carcass classes. Hatching success was measured by transferring clutches to agar plates and so was independent of the Full Care/No Care manipulations. The raw data and the means ±SE are displayed

The relationship between clutch size and carcass mass was similar to the results obtained in testing Prediction 2. In general, clutches were smaller when females were given smaller dead mice to breed upon, but No Care females breeding on a small carcass laid significantly fewer eggs than did Full Care females (Figure 3, Table 2).

For females breeding on a small carcass, we found that the hatching success of clutches laid by No Care females was significantly greater than those laid by Full Care females. For females breeding on medium or large carcasses, there was no difference in the hatchability of eggs laid by No Care and Full Care females (Figure 3, Table 2). For these females, there was an overall positive relationship between clutch size and hatching success, but it was not statistically significant (Table 2).

**TABLE 2:** Estimates from models on the hatching success and the sizes of clutches laid by Full Care (FC) and No Care (NC) females across three different carcass classes: small (8–9 g), medium (16–17 g), or large (23–24 g). (a) A generalized linear model of the proportion of eggs that successfully hatched. (b) A linear model of the clutch size

(a) Hatching success	Estimate	Std error	*z* Value	*p*
Intercept	1.3629	0.1811	**7.524**	**<.001**
Population (NC)	1.0283	0.1881	**5.466**	**<.001**
Carcass class (Med)	0.1345	0.1358	0.991	.322
Carcass class (Large)	−.0768	0.1319	−0.582	.560
Clutch size	.0088	.0048	1.810	.070
Population (NC) × Carcass class (Med)	−1.0547	0.2313	**−4.560**	**<.001**
Population (NC) × Carcass class (Large)	−0.9340	0.2242	**−4.165**	**<.001**
Block (2)	0.2349	.0837	**2.807**	**.005**

(b) Clutch size	Estimate	Std error	*t* Value	*p*
Intercept	−10.294	7.573	−1.359	.176
Population (NC)	−5.856	2.142	**−2.734**	.**007**
Carcass class (Med)	1.069	2.097	0.510	.611
Carcass class (Large)	5.029	2.113	**2.381**	.**018**
Female size	8.713	1.616	**5.392**	**<.001**
Population (NC) × Carcass class (Med)	9.340	2.952	**3.164**	.**002**
Population (NC) × Carcass class (Large)	8.127	2.975	**2.732**	.**007**
Block (2)	5.605	1.215	**4.612**	**<.001**

Note

Sample sizes were: 42 Full Care and 45 No Care in Block 1; 45 Full Care and 43 No Care in Block 2. Significant terms are in bold.

## 4 DISCUSSION

Our results suggest that the evolution of an “optimistic” clutch size, and the overproduction of offspring, critically depends on a mechanism for killing surplus offspring (Forbes & Mock, 1998; Houston et al., 2012) rather than on uncertainty about prospects for reproductive success. In a No Care environment, where partial filial cannibalism after hatching was not possible, selection favored a reduction in clutch size on a smaller carcass because it resulted in the production of heavier larvae, each with greater future fitness prospects (Bladon et al., 2020). Consequently, after experimental evolution, No Care females laid fewer eggs when given a small mouse to breed upon than their counterparts in the Full Care populations, where partial filial cannibalism was still possible. We found a correlated change in hatching success too: The production of a more restrained clutch size on small carrion by the No Care populations was associated with greater hatching success. However, we found no difference in the clutch sizes produced when No Care and Full Care females were given larger carrion to breed upon. This could be because we did not use carrion of this size in generating the evolved populations and so they were not divergently adapted for breeding on mice this large. In addition, or instead, it could be that the costs to the No Care females of producing larger clutches are negligible when larvae have plentiful resources on medium and large carrion, since there is sufficient food for all larvae to attain a relatively high mass by the time they disperse away from the carcass.

This interpretation of the data makes three assumptions: (1) that the divergence we describe between Full Care and No Care populations is due to evolved change; (2) that it is the No Care populations that have evolved, whereas the Full Care populations are more representative of the ancestral wild populations; and (3) that the Full Cares are still overproducing offspring, whereas the No Cares are not. We think each of these assumptions is likely to be true, for the following reasons.

We attribute our results to evolved change, rather than parental effects alone, because our analyses indicate that selection favors a reduction in clutch size in a No Care environment. Furthermore, in previous work, conducted after 6 generations of experimental evolution, we passed the Full Care and No Care populations through a common garden environment and found evidence of divergent local adaptation to the No Care and Full Care environments (Schrader et al., 2015b). After 11 generations of experimental evolution, we analyzed the number of eggs laid on small carrion by No Care and Full Care populations, again after passing them through a common garden environment. We found that No Care females consistently laid around four fewer eggs than Full Cares—in keeping with the results we report here (Duarte et al., 2021). In addition, the results we report here are not simply due to an intrinsic reduction in female quality in the No Care populations. When given larger carrion to breed upon, No Care females were still able to produce more eggs, and as many eggs, as females from Full Care populations in generation 20 and 22 (Figures 1 and 2). By generation 23 of experimental evolution, larvae attained a similar mass by the time they dispersed away from the carcass in both the Full Care and No Care populations (Rebar et al., 2020).

It is likely that our results are due to greater evolved change in the No Care populations than in the Full Care populations. In Sun et al. (2020), we describe the reaction norm linking clutch size to carcass size in two of the wild populations that contributed to the founding population for the experimental populations we studied here. We found a decrease in clutch size of roughly 2–3 eggs over an approximately 12 g decrease in carrion mass. We did not formally derive equivalent reactions norms for the populations studied here. Nevertheless, the Full Care populations responded similarly to the wild populations, showing a decrease of 1–2 eggs over a 16 g decrease in carrion mass. By contrast, the No Care populations showed a decrease of about 20 eggs over the same 16 g decrease in carrion mass.

Finally, after 22 generations of breeding on an identical range of mouse carrion (8–14 g), in an identical laboratory environment, the Full Care and No Care populations converged on producing a similar number of larvae at dispersal (Schrader et al., 2017), which attained a similar mass at dispersal (Rebar et al., 2020)—a key correlate of fitness in burying beetles (Lock et al., 2004; Pascoal et al., 2018). Nevertheless, we have shown here (and elsewhere; Duarte et al., 2021) that Full Care populations lay more eggs than the No Cares do, presumably because they can still cull any surplus young after hatching.

We cannot tell from our data exactly how females in the No Care populations have evolved to be better at matching their clutch size to small carrion, but two factors may have contributed to different degrees. Previous work has shown that the number of eggs laid by female burying beetles depends partly on female size, partly on carcass size, and partly on the size of the first male to mate with the female (Pascoal et al., 2018). It is possible that the relative influence of these different contributing factors has evolved to be different in the No Care populations such that the clutch size laid by females from these populations is now more strongly influenced by carcass size than by their own size.

Alternatively, or as well, No Care females may have evolved to be better able to estimate the resources on a small dead body that can be used for reproduction. Previous work on *Nicrophorus* burying beetles has shown that females use volume, rather than mass, to assess resource availability and then determine the number of eggs to lay (Trumbo & Fernandez, 1995). It is possible that females estimate resource availability from the ball of flesh they make from the dead body, which becomes the edible nest for their larvae. We have suggested before that a rounder carcass could provide a female with more accurate information about its volume, allowing her to make more fine‐scaled adjustments in clutch size (De Gasperin et al., 2016). We have also found that No Care parents have evolved to invest more in carcass preparation than Full Care parents (Jarrett, Evans, et al., 2018) and make nests that are more spherical than those made by Full Care parents (Duarte et al., 2021), despite the greater fitness costs associated with building a rounder nest (De Gasperin et al., 2016). Perhaps this change in nest construction enables No Care females to more accurately assess the resources available for nourishing their brood.

Our results imply that the wild populations from which we sourced our experimental populations have considerable standing genetic variation in the shape of the reaction norm linking carrion size to clutch size. The data collected from the Full Care populations imply that much of this genetic variation is expressed as variation in clutch sizes across carcass sizes (Figure 1). We suggest that a high level of genetic variation is due directly to partial filial cannibalism because it relaxes selection for a more precise match between carrion size and clutch size, especially on smaller carrion. We predict flatter reaction norms (i.e., slopes approaching 0) in other species where partial filial cannibalism is expressed because recouping some energy through offspring consumption lowers the costs of overproduction.

The experiment testing Prediction 3 suggests that the overproduction of offspring in turn relaxes selection on egg hatchability—at least when females breed on small carcasses. Under these conditions, females from the No Care populations not only produced fewer eggs but laid eggs that were more likely to hatch (Figure 2). We do not yet know why the eggs laid by No Care females had greater hatchability on small carcasses, though it may be connected to a trade‐off between egg size and clutch size. This could also explain why hatchability was not improved in the No Care populations when they laid more eggs on medium and large carcasses: In these conditions, there are no additional resources for enhancing egg size and hence hatchability. Alternatively, or in tandem, the costs of having too few eggs hatch are most costly on small carcasses for females from the No Care populations. If only a few larvae from a clutch hatch, they may not be able to penetrate the carcass resources and thus the entire brood fails (Jarrett, Rebar, et al., 2018).

Our findings relate to the extensive wider literature on the evolution of clutch size in three different ways. First, whereas previous work has emphasized the importance of parental uncertainty in driving the overproduction of offspring (Forbes, 1991, 1997; Kozlowski & Stearns, 1989; Lack, 1947; Magrath, 1989; Mock & Forbes, 1995; Mock & Parker, 1997; Ricklefs, 1965; Temme & Charnov, 1987), relatively little experimental work has analyzed the importance of an efficient mechanism for dispensing with surplus offspring. Here we have shown that the overproduction of offspring ceases when such a mechanism is eliminated experimentally, presumably because the net fitness benefits are then substantially reduced. Under these circumstances, females evolve to lay a more prudent clutch size instead.

Second, in common with previous work on other species (reviewed by Kilner & Hinde, 2012), we found that selection acts through parents to favor the production of more offspring, but acts through offspring fitness to favor the production of fewer, smaller offspring. Our experiments suggest that when parents have a mechanism for efficiently dispensing with surplus offspring, selection acting on parents favors the evolution of larger clutches. However, when that mechanism is eliminated, selection acting on offspring favors a reduction in clutch size. More generally, our results imply that any parent‐offspring conflict over initial family size will be resolved in the parents’ favor while there is a mechanism for killing surplus offspring after birth—whether that mechanism involves partial filial cannibalism, other forms of infanticide, or the parental incitement of siblicide (Mock & Parker, 1997).

Finally, our experimental results show that the overproduction of offspring can relax selection against low egg hatchability, particularly when resources are limiting. The novel insight here is that the two traits are thus mutually reinforcing. Low egg hatchability selects for the overproduction of offspring, but the overproduction of offspring causes hatching failures to persist.

## CONFLICT OF INTEREST

The authors declare no conflict of interest.

## AUTHOR CONTRIBUTIONS

**Darren Rebar:** Conceptualization (equal); Data curation (lead); Formal analysis (lead); Investigation (equal); Methodology (equal); Project administration (equal); Supervision (equal); Writing – original draft (lead); Writing – review & editing (equal). **Chay Halliwell:** Data curation (supporting); Investigation (supporting); Methodology (supporting); Project administration (supporting); Writing – review & editing (supporting). **Rachel Kemp:** Data curation (supporting); Investigation (supporting); Methodology (supporting); Project administration (supporting); Writing – review & editing (supporting). **Rebecca M. Kilner:** Conceptualization (equal); Formal analysis (supporting); Funding acquisition (lead); Investigation (equal); Methodology (equal); Supervision (equal); Writing – original draft (supporting); Writing – review & editing (equal).

## Supporting information

Figure S1

Figure S2

Figure S3

Supplementary Material

## Data Availability

Data supporting the findings are available in DRYAD (https://doi.org/10.5061/dryad.v15dv41z0).
