# Supplementary figures and images for: Experimental evolution of a more restrained clutch size when filial cannibalism is prevented in burying beetles Nicrophorus vespilloides
Source: Ecol Evol. 2022 Apr 15;12(4):e8829. doi: 10.1002/ece3.8829 (PMC9012908; doi:10.1002/ece3.8829)

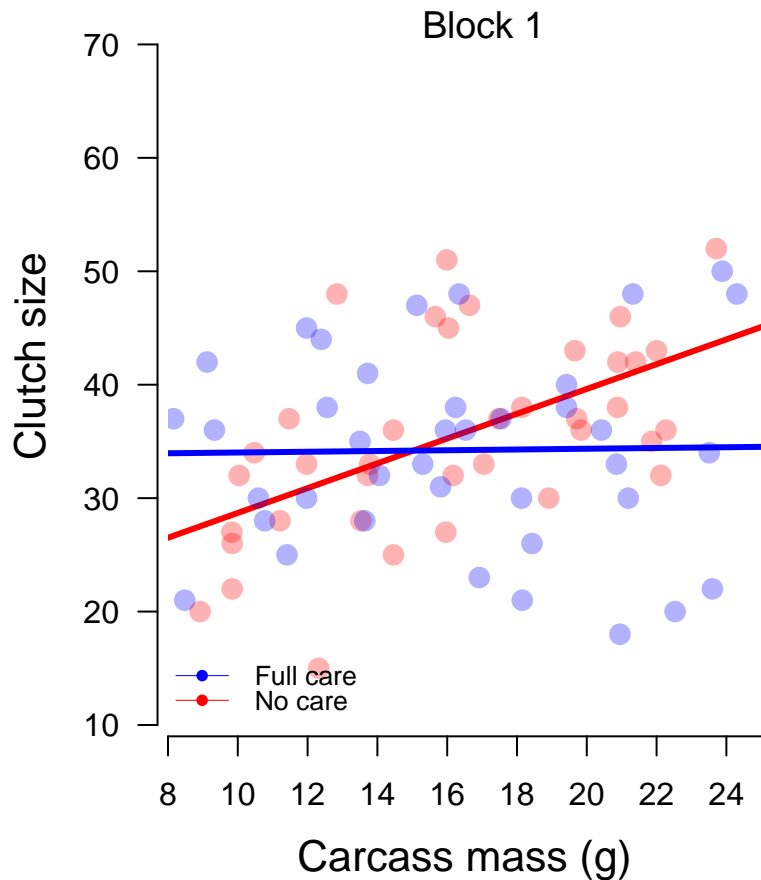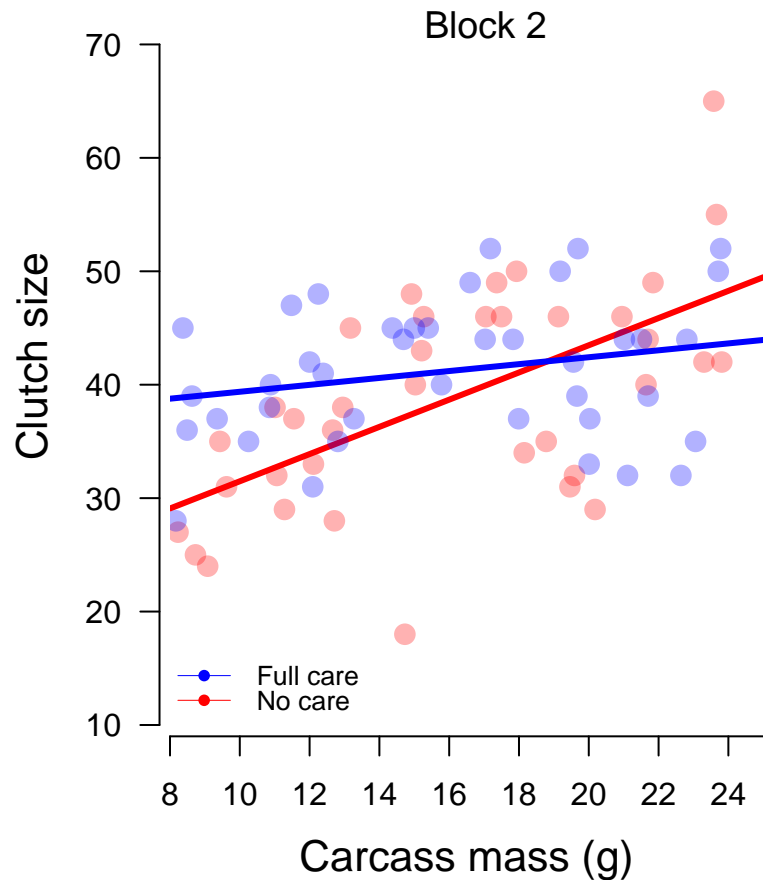

Supplement: Supplementary file 1 — Figure S1 [file ECE3-12-e8829-s001.pdf]

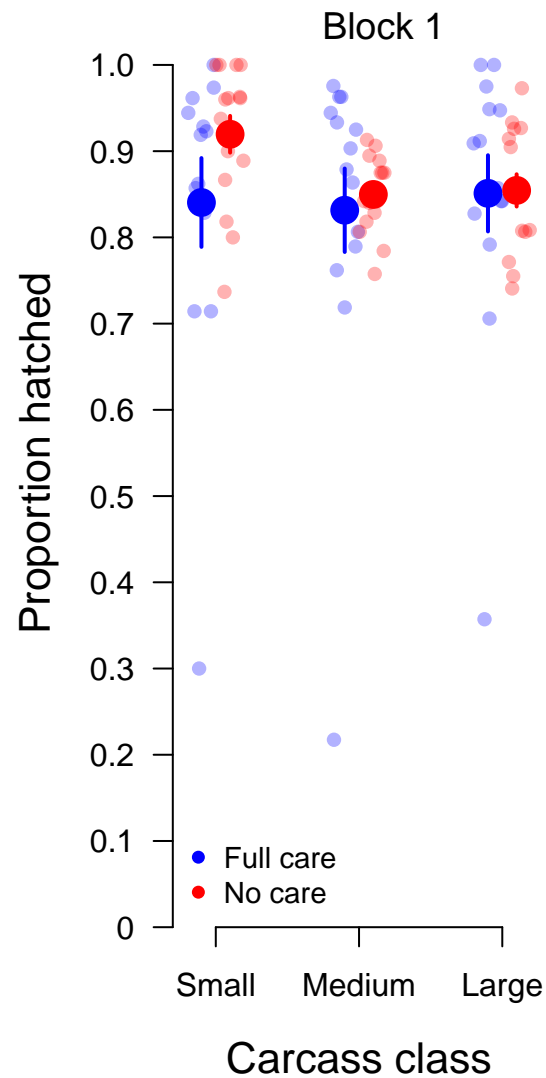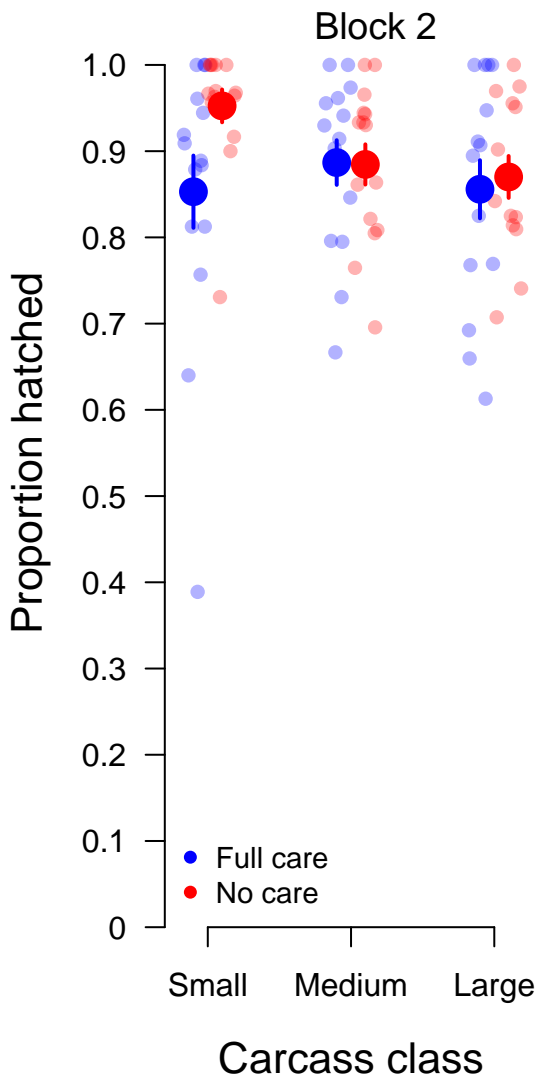

Supplement: Supplementary file 2 — Figure S2 [file ECE3-12-e8829-s002.pdf]

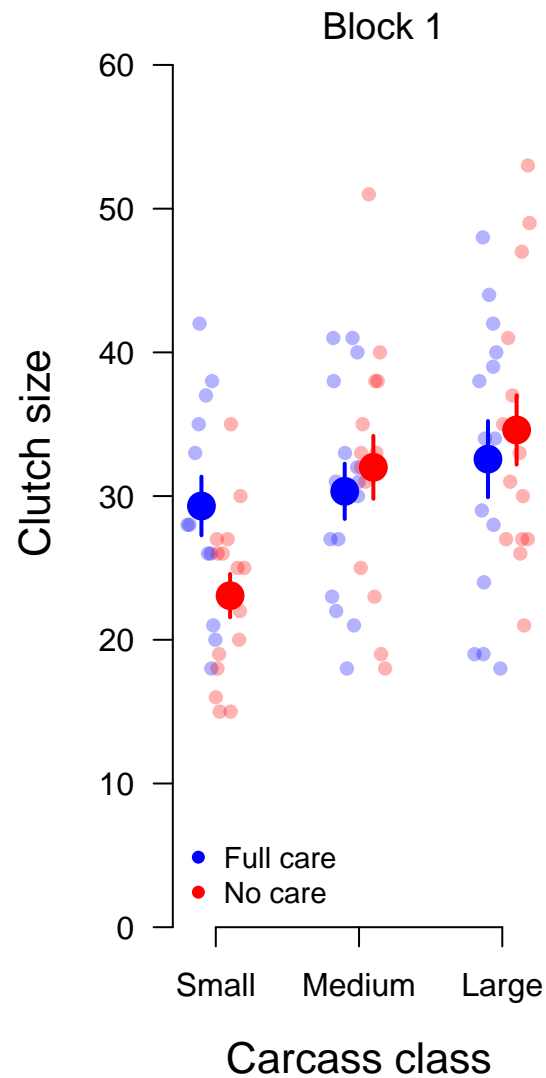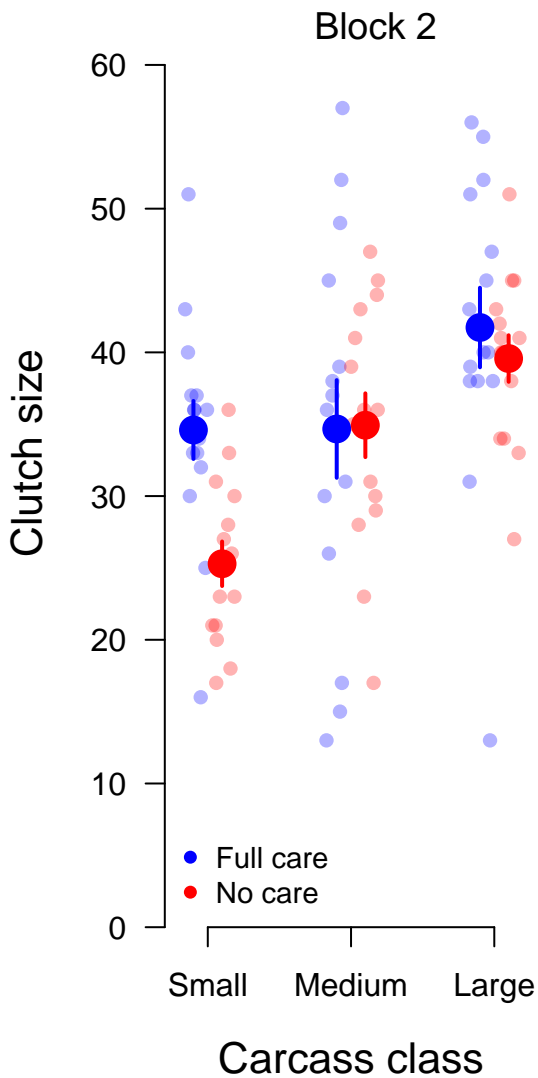

Supplement: Supplementary file 3 — Figure S3 [file ECE3-12-e8829-s004.pdf]
