# Supplementary material for: Experimental evolution of a more restrained clutch size when filial cannibalism is prevented in burying beetles Nicrophorus vespilloides
Source: Ecol Evol. 2022 Apr 15;12(4):e8829. doi: 10.1002/ece3.8829 (PMC9012908; doi:10.1002/ece3.8829)
Supplement: Supplementary file 4 — Supplementary Material [file ECE3-12-e8829-s003.docx]

APPENDIX A

**Table A1.** Estimates of linear models analyzing the effect of carcass mass on the size of clutches laid by Full Care (FC) and No Care (NC) females, run independently for each pair of FC and NC replicated populations (i.e., Block in Table 1). Significant terms are bolded.

| **CLUTCH SIZE (Block 1)** | **Estimate** | **Std error** | **t value** | ***p*** |
| --- | --- | --- | --- | --- |
| Intercept | -7.6226 | 11.2353 | -0.678 | 0.500 |
| Population (NC) | -8.5679 | 6.5810 | -1.302 | 0.197 |
| Carcass mass | 0.0924 | 0.2564 | 0.360 | 0.720 |
| Population (NC) × Carcass mass | 0.7642 | 0.3840 | **1.990** | **0.050** |
| Female size | 8.5964 | 2.1580 | **3.983** | **<0.001** |

| **CLUTCH SIZE (Block 2)** | **Estimate** | **Std error** | **t value** | ***p*** |
| --- | --- | --- | --- | --- |
| Intercept | 14.3036 | 9.3110 | 1.536 | 0.129 |
| Population (NC) | -14.4930 | 5.4836 | **-2.643** | **0.010** |
| Carcass mass | 0.2656 | 0.2217 | 1.198 | 0.235 |
| Population (NC) × Carcass mass | 0.8050 | 0.3246 | **2.480** | **0.016** |
| Female size | 4.8325 | 1.8735 | **2.579** | **0.012** |

**Table A2.** Estimates of generalized linear models analyzing potential predictors of the proportion of eggs from clutches laid by Full Care (FC) and No Care (NC) females that successfully hatched, across three different carcass size classes. Models were run separately for each block. Significant terms are bolded.

| **HATCHING SUCCESS Block 1** | **Estimate** | **Std error** | **z value** | ***p*** |
| --- | --- | --- | --- | --- |
| Intercept | 1.8214 | 0.2588 | **7.039** | **<0.001** |
| Population (NC) | 0.7940 | 0.2566 | **3.094** | **0.002** |
| Carcass class (Med) | -0.0636 | 0.1928 | -0.330 | 0.742 |
| Carcass class (Large) | -0.0845 | 0.1941 | -0.435 | 0.663 |
| Clutch size | -0.0027 | 0.0069 | -0.390 | 0.697 |
| Population (NC) × Carcass class (Med) | -0.7574 | 0.3160 | **-2.397** | **0.017** |
| Population (NC) × Carcass class (Large) | -0.6835 | 0.3136 | **-2.179** | **0.029** |

| **HATCHING SUCCESS Block 2** | **Estimate** | **Std error** | **z value** | ***p*** |
| --- | --- | --- | --- | --- |
| Intercept | 1.0804 | 0.2758 | **3.917** | **<0.001** |
| Population (NC) | 1.3014 | 0.2812 | 4.628 | <0.001 |
| Carcass class (Med) | 0.3227 | 0.1944 | 1.661 | 0.097 |
| Carcass class (Large) | -0.1249 | 0.1810 | -0.690 | 0.490 |
| Clutch size | 0.0208 | 0.0069 | **3.015** | **0.003** |
| Population (NC) × Carcass class (Med) | -1.3584 | 0.3455 | **-3.932** | **<0.001** |
| Population (NC) × Carcass class (Large) | -1.1746 | 0.3265 | **-3.597** | **<0.001** |

**Table A3.** Estimates of a linear model analyzing predictors of the sizes of clutches laid across three different carcass size classes by Full Care (FC) and No Care (NC) females. Models were run separately for each block. Significant and marginally significant terms are bolded.

| **CLUTCH SIZE Block 1** | **Estimate** | **Std error** | **t value** | ***p*** |
| --- | --- | --- | --- | --- |
| Intercept | -4.580 | 10.977 | -0.417 | 0.678 |
| Population (NC) | -3.899 | 3.029 | -1.287 | 0.202 |
| Carcass class (Med) | 1.328 | 2.938 | 0.452 | 0.652 |
| Carcass class (Large) | 3.310 | 1.985 | 1.109 | 0.271 |
| Female size | 7.353 | 2.336 | **3.148** | **0.002** |
| Population (NC) × Carcass class (Med) | 8.105 | 4.079 | **1.987** | **0.050** |
| Population (NC) × Carcass class (Large) | 8.081 | 4.114 | **1.964** | **0.053** |

| **CLUTCH SIZE Block 2** | **Estimate** | **Std error** | **t value** | ***p*** |
| --- | --- | --- | --- | --- |
| Intercept | -10.963 | 10.354 | -1.059 | 0.293 |
| Population (NC) | -8.126 | 3.067 | **-2.650** | **0.001** |
| Carcass class (Med) | 0.960 | 3.009 | 0.319 | 0.751 |
| Carcass class (Large) | 6.581 | 3.005 | **2.190** | **0.031** |
| Female size | 10.230 | 2.275 | **4.496** | **<0.001** |
| Population (NC) × Carcass class (Med) | 10.773 | 4.292 | **2.510** | **0.014** |
| Population (NC) × Carcass class (Large) | 8.633 | 4.334 | **1.992** | **0.050** |

**Figure A1.** The relationship between clutch size and carcass mass for females who evolved in the Full Care (blue) and No Care (red) experimental populations, separated by block. Lines are the linear regression lines for each population.

**Figure A2.** The proportion of eggs that hatched from clutches laid by Full Care (blue) and No Care (red) females across three carcass classes, separated by block. The raw data and the means ± SE are displayed.

**Figure A3.** The size of clutches laid by Full Care (blue) and No Care (red) females across three carcass classes, separated by block. The raw data and the means ± SE are displayed.
